# Thioredoxin Reductase Deficiency Potentiates Oxidative Stress, Mitochondrial Dysfunction and Cell Death in Dopaminergic Cells

**DOI:** 10.1371/journal.pone.0050683

**Published:** 2012-11-30

**Authors:** Pamela Lopert, Brian J. Day, Manisha Patel

**Affiliations:** 1 Neuroscience Program, University of Colorado Anschutz Medical Campus, Aurora, Colorado, United States of America; 2 Department of Pharmaceutical Sciences, University of Colorado Anschutz Medical Campus, Aurora, Colorado, United States of America; 3 Department of Medicine, National Jewish Health, Denver, Colorado, United States of America; Emory University, United States of America

## Abstract

Mitochondria are considered major generators of cellular reactive oxygen species (ROS) which are implicated in the pathogenesis of neurodegenerative diseases such as Parkinson’s disease (PD). We have recently shown that isolated mitochondria consume hydrogen peroxide (H_2_O_2_) in a substrate- and respiration-dependent manner predominantly via the thioredoxin/peroxiredoxin (Trx/Prx) system. The goal of this study was to determine the role of Trx/Prx system in dopaminergic cell death. We asked if pharmacological and lentiviral inhibition of the Trx/Prx system sensitized dopaminergic cells to mitochondrial dysfunction, increased steady-state H_2_O_2_ levels and death in response to toxicants implicated in PD. Incubation of N27 dopaminergic cells or primary rat mesencephalic cultures with the Trx reductase (TrxR) inhibitor auranofin in the presence of sub-toxic concentrations of parkinsonian toxicants paraquat; PQ or 6-hydroxydopamine; 6OHDA (for N27 cells) resulted in a synergistic increase in H_2_O_2_ levels and subsequent cell death. shRNA targeting the mitochondrial thioredoxin reductase (TrxR2) in N27 cells confirmed the effects of pharmacological inhibition. A synergistic decrease in maximal and reserve respiratory capacity was observed in auranofin treated cells and TrxR2 deficient cells following incubation with PQ or 6OHDA. Additionally, TrxR2 deficient cells showed decreased basal mitochondrial oxygen consumption rates. These data demonstrate that inhibition of the mitochondrial Trx/Prx system sensitizes dopaminergic cells to mitochondrial dysfunction, increased steady-state H_2_O_2_, and cell death. Therefore, in addition to their role in the production of cellular H_2_O_2_ the mitochondrial Trx/Prx system serve as a major sink for cellular H_2_O_2_ and its disruption may contribute to dopaminergic pathology associated with PD.

## Introduction

Mitochondrial reactive oxygen species (ROS) play important roles in cell signaling as well as pathological processes including oxidative damage in neurodegenerative disorders such as Parkinson’s disease (PD) [1]–[3]. Mitochondria are known to be major generators of ROS which includes superoxide (O_2_^.-^), hydrogen peroxide (H_2_O_2_), and hydroxyl radicals (HO^.^) [4]. To maintain the delicate balance of ROS production (for signaling pathways) and consumption (to prevent oxidative damage), the mitochondria have multiple antioxidant pathways for ROS detoxification. Mitochondrial manganese superoxide dismutase (SOD2) converts the O_2_^.-^ radical to H_2_O_2_ which is then converted to water through the thioredoxin/peroxiredoxin (Trx/Prx) or the glutathione (GSH) pathway. Given the notable absence of catalase in brain mitochondria, the relative importance of the GSH and Trx/Prx enzymatic pathways in H_2_O_2_ consumption by brain mitochondria remains unknown. The Trx/Prx pathway detoxifies ROS through Prx converting H_2_O_2_ into water. Prx is kept in a reduced state by Trx which itself is kept in the reduced form through the action of thioredoxin reductase (TrxR) [5]. Recent studies suggest a crucial role for the mitochondrial thioredoxin reductase (TrxR2) i.e. deletion of TrxR2 renders mice embryonic lethal at day 13 and inhibition of TrxR2 in insolated heart mitochondria results in increased H_2_O_2_ emission [6], [7]. Using polarographic methods for real-time detection of steady state H_2_O_2_ levels, we recently demonstrated that brain mitochondria consume H_2_O_2_ in a respiration-dependent manner predominantly via the Trx/Prx system in comparison to the GSH system [8]. This study demonstrated that direct pharmacological inhibition of TrxR by auranofin (Aur) and Prx3 inhibition by phenethyl isothiocyanate attenuated H_2_O_2_ removal by 80% and 50%, respectively whereas the GSH pathway was only responsible for up to 15% of exogenous H_2_O_2_ removal by isolated brain mitochondria [8]. Moreover, brain mitochondria showed unique dependence on substrates and the Trx/Prx system compared to liver mitochondria [8]. Although these studies suggest a crucial role of Trx/Prx system in H_2_O_2_ consumption in brain mitochondria, the role of the mitochondrial Trx/Prx system and its contribution to neurodegeneration in conditions of enhanced oxidative stress is unknown.

We hypothesized that the mitochondrial Trx/Prx system is critical for maintenance of the redox status in neuronal cells under oxidative stress. Given the important role of oxidative stress and mitochondrial dysfunction in PD, in this study we sought to determine the significance of the mitochondrial Trx/Prx system in dopaminergic (DA) cells subjected to model toxicants implicated to cause parkinsonism e.g. paraquat (PQ) and 6-hydroxydopamine (6OHDA) [9]–[12]. Here we demonstrate that pharmacological inhibition of TrxR or lentiviral knock-down of TrxR2 sensitizes dopaminergic cells to sub-toxic concentrations of PD toxicants PQ and 6OHDA.

## Methods

### Chemical Reagents

Auranofin (S-triethylphosphinegold (I)-2,3,4,6-tetra-O-acetyl-1-thio-β-D-glucopyranoside) was obtained from Alexis Biochemicals (San Diego, CA, USA). Catalytic antioxidant manganoporphyrin AEOL 10150 [13] was provided by Aeolus Pharmaceuticals (Mission Viejo, CA, USA). All other chemicals and reagents were obtained from Sigma-Aldrich (St. Louis, MO, USA) unless otherwise noted.

### N27 Cell Culture

Immortalized rat dopaminergic N27 cells were a generous gift from Drs. Curt Freed and Kedar Prasad at the University of Colorado, Anschutz Medical Campus [14]. Cell culture reagents were obtained from Invitrogen (Carlsbad, CA, USA). N27 cells were grown and plated as previously reported [14], [15]. Briefly, cells were grown in RPMI 1640 medium supplemented with 10% heat-inactivated fetal bovine serum (FBS), penicillin (100 U/mL) and streptomycin (100 U/mL). Cells were plated for experimentation in RPMI 1640 supplemented with 1% FBS, penicillin (10 U/mL) and streptomycin (10 U/mL) and maintained at 37°C in a 5% CO_2_ humidified atmosphere. All experiments were conducted in cells passage #3–20.

### Primary Ventral Mesencephalic Cell Culture

Mixed neuronal and glial cultures were prepared as previously reported [16]. Cells were plated in pretreated poly-D-lysine coated 96-well, 12-well or 6-well plates. The medium was not replaced and all experiments were conducted in 2 week old (13–15 days) cells. Cells were maintained in 37°C in a 5% CO_2_ humidified atmosphere. Animal procedures have been reviewed and approved by the University of Colorado Anschutz Medical Campus Institutional Animal Care and Use Committee. The necessary care was taken to minimize any animal suffering and pain.

### Isolation of Mitochondria from Cell Culture

Mitochondria were isolated using the Mitochondria Isolation Kit for Cultured Cells (Thermo Scientific, Rockford, IL, USA) according to manufacturer’s instructions. Briefly, 2 x 10^7^ cells were harvested and subjected to isolation through the reagent-based method. Mitochondria were sonicated and protein levels were determined by a Bradford Protein assay (Thermo Scientific).

### TrxR2 Knockdown

TrxR2 was inhibited in N27 cells using SMART vector 2.0 lentiviral shRNA particles according to manufacturer’s protocol (Thermo Scientific Dharmacon, Lafayette, CO, USA). Briefly, N27 cells were plated in a 6-well plate with 160,000 cells/well in reduced serum media overnight. Cells were treated with 5 µg/mL polybrene (American Bioanalytical, Natick, MA, USA) in RPMI 1640 and transduced at a multiplicity of infection (MOI) of 20. Cells where transfected with either TrxR2 targeting (TrxR2 deficient) or non-targeting negative control particles (Mock). After 18 hrs, cells where washed and allowed to grow for 48–72 hrs. TrxR2 deficient and mock cells were selected using 10 µg/mL puromycin and cells were maintained with RPMI 1640 medium supplemented with 10 µg/mL puromycin, 10% heat-inactivated fetal bovine serum (FBS), penicillin (100 U/mL) and streptomycin (100 U/mL). Three predesigned individual gene specific shRNA lentiviral particles were obtained from Dharmacon and screened for transfection efficiency and the particle with the sequence AGTGCTAATAAAGAGCGTG was chosen. TrxR2 deficient cells had a similar morphology as mock controls however they appeared to grow at a slower rate. To address this issue, all experiment (passage #3–10) were normalized to protein levels.

### Verification of TrxR2 Deficient Cells

RNA from transfected N27 cells was isolated using the RNeasy kit® (Qiagen, Valencia, CA, USA) according to manufacturers protocol and quantified through 260/280 wavelength measurement. RNA was reverse transcribed using the high capacity cDNA reverse transcription kit according to manufactures protocol (Applied Biosystems, Foster City, CA, USA). Real time PCR was performed on an Applied Biosystems 7500 Fast Real-Time PCR system. Primers and probes for rat TrxR2 were purchased from Applied Biosystems.

### TrxR2 Activity Assay

TrxR activity was measured in cells and isolated mitochondria using an insulin-reduction assay in the presence of *E. coli* thioredoxin as previously described by Arnér et al. [5]. Cells were lysed by Tris/NaCl solution with 0.1% Triton-X and mitochondria were lysed using sonication. After 1 hr incubation the number of reduced thiols was determined on a Versamax micro plate reader (Molecular Devices, Sunnyvale, CA, USA). Protein levels were determined using a Bradford protein assay.

### Detection of H_2_O_2_

H_2_O_2_ was measured using the Amplex Red assay (Invitrogen) and conducted as outlined by Cantu et al [15]. Fluorescence was measured by a Synergy™ multi-mode microplate reader (Biotek, Winooski, VT, USA).

### Cell Death Assessment

Cell death was determined by measuring release of lactate dehydrogenase (LDH) enzyme activity as described by Bergmeyer et al [17] and published previously [18]. Cells were plated in 96-well plated at 2.0×10^4^ cells/well. Media and cell lysis (Tris/NaCl +0.1% Triton) samples were collected and LDH levels was measured.

### Polarographic Measurement of Exogenous H_2_O_2_ removal

H_2_O_2_ removal rates were measured in 1×10^6^ cells per sample using a 100-µM Clark-type electrode with an Apollo 4000 Free Radical Analyzer (World Precision Instruments, Inc., Sarasota FL.) as outlined by Drechsel et al [8]. Briefly, after obtaining a stable baseline (60–90 sec) 3 µM of exogenous H_2_O_2_ was added to an open, thermostatted chamber (30°C) and allowed to stabilize for 30–60 sec. 1×10^6^ cells were added to the chamber and H_2_O_2_ removal rates were calculated based on the linear signal decay after the addition of cells compared to H_2_O_2_ alone. Rates were normalized to protein levels as determined by a Bradford Protein Assay.

### Measurement of Oxygen Consumption Rate (OCR)

Oxygen consumption rates were determined using a Seahorse XF24 analyzer (Seahorse Biosciences, North Billerica, MA, USA) as previously reported by Cantu et al [15]. Briefly cells were plated at 30,000 cells/well and incubated overnight with serum-free RPMI. Different parameters of respiration were calculated by subtracting the average respiration rates before and after the addition of the electron transport inhibitors [0.1 uM Oligomycin, 0.3 uM cyanide-p-trifluoromethoxyphenylhydrazone (FCCP) and 0.3 uM Antimycin-A]. The parameters calculated included: basal respiration (baseline respiration minus antimycin-A post injection respiration), ATP turnover (baseline respiration minus oligomycin post injection respiration), H+ leak (oligomycin-respiration minus antimycin-A post injection respiration), maximal respiratory capacity (FCCP stimulated respiration minus antimycin-A post injection respiration) and reserve respiratory capacity (FCCP stimulated respiration minus baseline respiration).

### Western Blot

Detection of Complex IV (Cox IV) was conducted as previously described in Cantu et al [15]. Briefly, cells were lysed and sonicated and protein levels were measured via Bradford protein assay. 10–15 µg of protein was loaded on a 12% gel (Bio-Rad, Hercules, CA, USA) and proteins were detected with a mouse monoclonal antibody (1∶500 Mitosciences, Eugene, OR, USA). A rabbit affinity-purified against rat β-actin secondary (1∶5,000 Sigma-Aldrich) was used to confirm equal loading in gels. Blots were analyzed using densitometry with ImageJ (NIH, Bethesda, MD, USA) and Cox IV was normalized to β-actin levels.

### ATP Levels

ATP levels were determined utilizing the ATP determination kit from Invitrogen according to manufactures protocol using 50,000 cells/well in 96-well plate. All levels were normalized to protein levels determined by a Bradford protein assay.

### Statistical Methods

Data were analyzed in GraphPad Prism software (version 5). Two-way ANOVA was used to test differences between Aur ± PQ with a Bonferroni post-test. To determine statistical significance between the same cell type with varying concentrations of the same compound a 1-way ANOVA with a Tukey’s multiple comparison test was utilized. Finally, to determine changes between the same treatment in different cell types a two-tailed student t-test was used.

## Results

### Consequences of Pharmacological Inhibition of TrxR in Primary Mesencephalic Cultures

**Figure 1:**
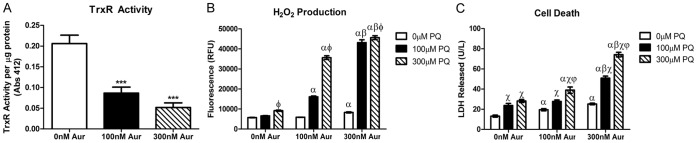
Pharmacological inhibition of TrxR in primary mesencephalic cultures results in decreased TrxR activity, increased H_2_O_2_ production and cell death. (a) Aur (100 and 300 nM) decreases the activity of TrxR after 6 hr of incubation *** =  p<0.0001 as determined by 1-way ANOVA (n = 10−18). (b) Subtoxic concentrations of Aur or PQ alone caused minimal increases in H_2_O_2_ production after 24 hrs. Aur and PQ caused a synergistic increase in H_2_O_2_ production (n  = 4−6). (c) After 48 hrs of treatment there was minimal cell death into the media in either treatment alone. Aur and PQ caused an additive effect on cell death (n  = 12−16). Bars represent mean ± SEM. α =  p<0.05 compared to 0 nM Aur in same PQ treatment, β = p<0.05 compared to 100 nM Aur in same PQ treatment, χ = p<0.05 compared to 0 µM PQ in same Aur treatment, φ = p<0.01 compared to 100 µM PQ in same Aur treatment as determined by 2-way ANOVA.

The role of the Trx/Prx system was determined in a cell-based model of oxidative stress. TrxR was pharmacologically inhibited using Aur which competitively binds the reduced selenocysteine located within the enzyme redox center [19]. Primary mesencephalic cultures were utilized which are a mixed astrocyte and neuronal culture [16]. Incubation of primary mesencephalic cultures for 6 hrs with Aur resulted in a significant decrease of TrxR activity to 63±7.6% at 100 nM and to 25±5.4% at the 300 nM concentration (Fig. 1a). We next determined if inhibition of the Trx/Prx system by Aur caused an increase in ROS production in the presence or absence of sub-toxic concentrations of the redox cycling agent PQ. ROS production was assessed using a fluorometric method which measures extracellular release of H_2_O_2_ through a horseradish peroxidase linked Amplex Red fluorescence assay [11]. As shown in figure 1b, incubation of primary mesencephalic cultures with either Aur or PQ alone for 24 hrs resulted in minor increases in H_2_O_2_ release (300 µM PQ alone and 300 nM Aur alone p<0.05). However, treatment of cultures with the combination of PQ and Aur resulted in a synergistic increase in H_2_O_2_ levels (p<0.001). This was observed in combined treatment with all concentrations of PQ and Aur. Finally, to determine whether the effect of increased H_2_O_2_ levels resulted in cell death, cultures were incubated with PQ and Aur for 48 hrs and cell death was measured by release of lactate dehydrogenase (LDH) in the media [17]. As indicated in figure S1a, 24 hr incubation of primary cultures resulted in a ∼30–60% increase and a ∼10–40% increase in LDH release for Aur and PQ alone. With combined treatment, a 72 and 169% increase of LDH release with 100 µM PQ and 100 and 300 nM Aur, respectively and a 134 and 211% increase with 300 µM PQ and 100 and 300 nm Aur, respectively was observed. As indicated by figure 1c, when primary cultures were incubated for 48 hrs with either Aur or PQ alone there was a moderate increase of LDH released (∼50–90% for Aur alone and ∼80–150% increase for PQ treatment alone). However, incubation of cultures with both Aur and PQ resulted in an additive release of LDH after 48 hrs of treatment (287 and 463% increase of LDH released with 300 nM Aur and 100 or 300 µM PQ treatment respectively). The increased LDH release in mesencephalic cultures at 48 hrs was more robust than 24 hr and corresponded with the prolonged exposure to the synergistically increased H_2_O_2_ levels.

### Consequences of Pharmacological Inhibition of TrxR in N27dopaminergic Cells

**Figure 2:**
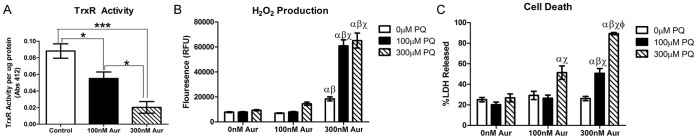
Pharmacological inhibition of TrxR in N27 cells results in decreased TrxR activity and increased H_2_O_2_ production and cell death. (a) Aur (100 and 300 nM) decreases the activity of TrxR after 6 hr of incubation in a concentration-dependent manner. * = p<0.005 *** =  p<0.0001 by 1-way ANOVA (n = 8−12). Subtoxic concentrations of Aur or PQ alone caused minimal increases in H_2_O_2_ production after 24 hrs (b) and cell death after 48 hrs (c). Aur and PQ caused a synergistic increase in H_2_O_2_ production and an additive effect on cell death. Bars represent mean ± SEM. α = p<0.05 compared to 0 nM Aur in same PQ treatment, β = p<0.05 compared to 100 nM Aur in same PQ treatment, χ = p<0.05 compared to 0 µM PQ in same Aur treatment,φ = p<0.01 compared to 100 µM PQ in same Aur treatment by 2-way ANOVA (n = 10−16).

Based on the results obtained in mesencephalic cultures, the next step was to determine the consequences of pharmacological inhibition of TrxR and knockdown of the mitochondria-specific TrxR. To achieve these goals, immortalized rat dopaminergic N27 cells were chosen. Incubation of N27 cells with 100 and 300 nM Aur resulted in a concentration-dependent inhibition of TrxR by ∼50% and ∼80%, respectively after 6 hrs (Fig. 2a).

In subsequent studies N27 cells were incubated with 100 nM and 300 nM Aur in the presence or absence of sub-toxic concentrations of PQ and H_2_O_2_ production (Fig. 2b) and cell death (Fig. 2c) were assessed at 24 and 48 hrs, respectively. In a manner similar to the primary mesencephalic cultures, Aur or PQ alone produced a minor increase in H_2_O_2_ release at the higher concentrations (300 µM PQ and 300nM Aur) and the combined treatment resulted in a synergistic increase (Fig. 2b). Additionally, after 48 hrs LDH release was potentiated in N27 cells incubated with both 300 µM PQ and 100 or 300 nM Aur (Fig. 2c). Each treatment alone caused a ∼20% release of total LDH which was similar in magnitude as control cells. However after 48 hrs of incubation with 100 nM Aur and 300 µM PQ, 50% of total LDH was released into the media and treatment with 300 nM Aur and 100 µM or 300 µM PQ resulted in 90% of total LDH released (Fig. 2c). As indicated in figure S1b, 24 hr incubation resulted in significant increases in %LDH released with the highest concentrations of agents in combination (300 nM Aur +100 or 300 µM PQ). Thus when cells are under sub-toxic oxidative stress, concomitant with inhibition of TrxR, a potentiation of H_2_O_2_ release and cell death occurs indicating the importance of the Trx/Prx pathway in H_2_O_2_ detoxification in both primary mesencephalic cultures and in a dopaminergic cell line.

### shRNA Treatment of N27 Cells Decreases Mitochondrial Specific TrxR mRNA and Activity

**Figure 3:**
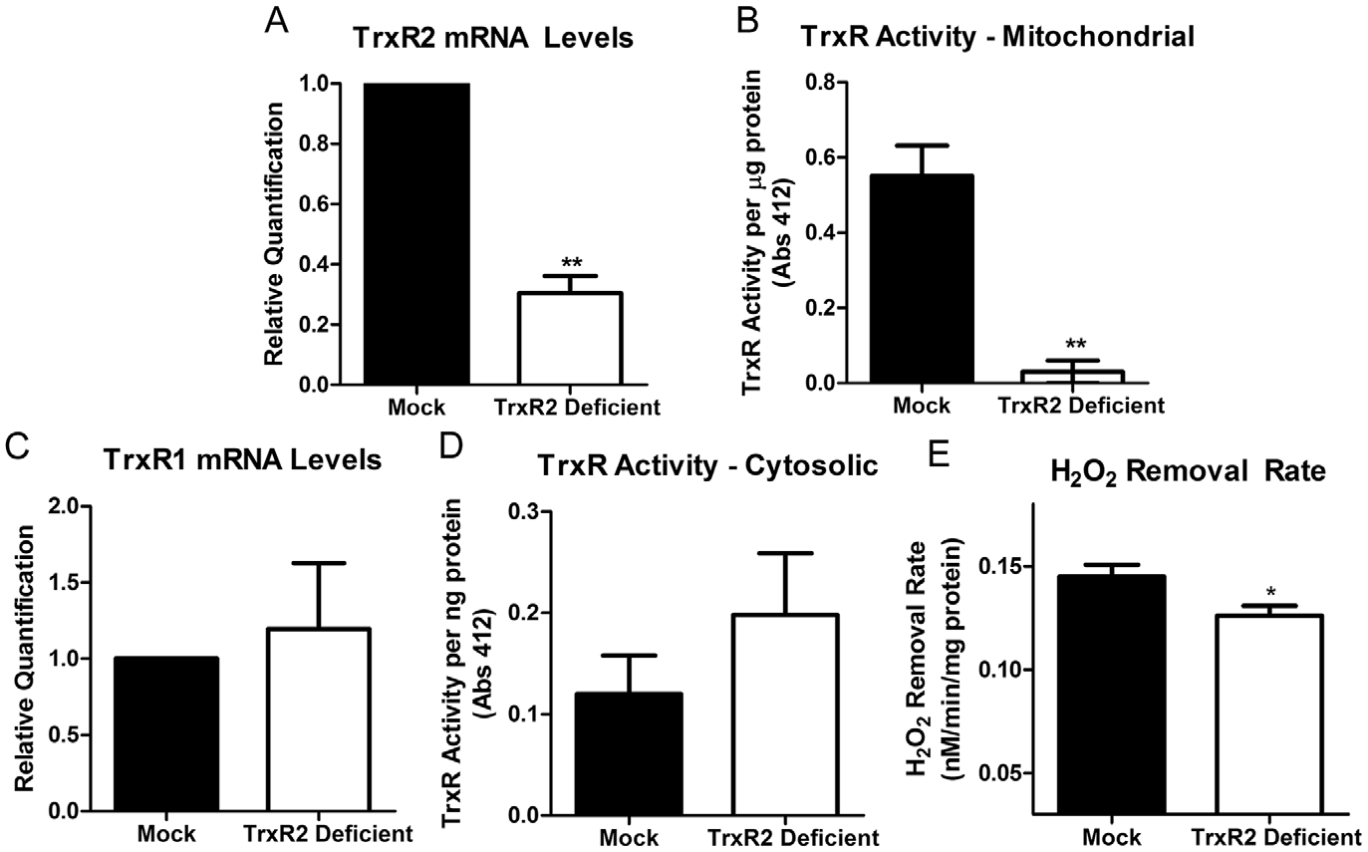
Generation of TrxR2 deficiency in N27 Cells. N27 cells were transfected with TrxR2 shRNA (TrxR2 deficient) and compared to mock-transfected cells (mock). (a) TrxR2 mRNA expression was measure by real-time PCR. Cells transfected with TrxR2 shRNA had a ∼60% decrease in TrxR2 mRNA compared to mock transfected cells (n = 3−6). (b) TrxR activity was measured in isolated mitochondria from mock and TrxR2 shRNA cells and there was a ∼95% loss in TrxR2 activity in the deficient vs. mock transfected cells (n = 3−6). To determine if the shRNA effect was mitochondrial specific TrxR1 mRNA (c) and TrxR activity (d) was measured in cytosolic fractions. There was no change in TrxR1 mRNA levels or activity (n = 2−6). (e) Mock and TrxR2 deficient cells were exposed to 3 µM exogenous H_2_O_2_ and the removal rates were determined with a Clark-type electrode. There was a significant (p<0.001) decrease in the TrxR2 deficient cells ability to remove exogenous H_2_O_2_ compared to mock controls (n = 9) Bars represent mean ± SEM. * =  <0.05, ** = p<0.005 as determined by two-tailed t-test.

As demonstrated in figures 1 and 2, inhibition of TrxR using the pharmacological compound Aur combined with PQ treatment produces synergistic H_2_O_2_ release compared to either agent alone. This data demonstrates the important role of H_2_O_2_ consumption via the Trx/Prx system under oxidative stress. Since TrxR has cyotosolic- (TrxR1) and mitochondrial (TrxR2) specific isoforms [20] and Aur does not demonstrate specificity towards a specific isoform, we sought to investigate the role of the mitochondria specific Trx/Prx pathway. shRNA constructs targeted to the mitochondria specific isoform were used to achieve stable knockdown of TrxR2 in N27 cells (Fig. 3). TrxR2 mRNA expression was measured in stably-transfected cells (passage 3–9) using real-time PCR and compared to mock transfected cells. As outlined in figure 3a there was a ∼70% decrease in TrxR2 mRNA levels in the TrxR2 transfected cells compared to mock transfected cells. The decreased mRNA correlated with a ∼95% decrease in TrxR2 activity in isolated mitochondria (Fig. 3b). To ensure the knockdown was mitochondria specific, mRNA levels of mock and shRNA transfected N27 cells were measured for the cytosolic isoform, TrxR1. As indicated in figure 3c there was no change in mRNA levels between transfection groups (Fig. 3c). Additionally, there was no change in TrxR activity in the cytosol of mock and TrxR2 deficient N27 cells (Fig. 3d). Finally, we previously showed that Aur inhibited H_2_O_2_ consumption in N27 cells by ∼36% [8]. Specific elimination of the mitochondrial TrxR2 resulted in a smaller albeit statistically significant decrease in H_2_O_2_ removal rates compared to mock transfected controls (Fig. 3e). Therefore, the shRNA approach was successful in knocking down mitochondrial specific TrxR levels and H_2_O_2_ consumption in N27 cells.

### TrxR2 Deficiency Sensitizes N27 Cells to H_2_O_2_ Release and Cell Death

**Figure 4:**
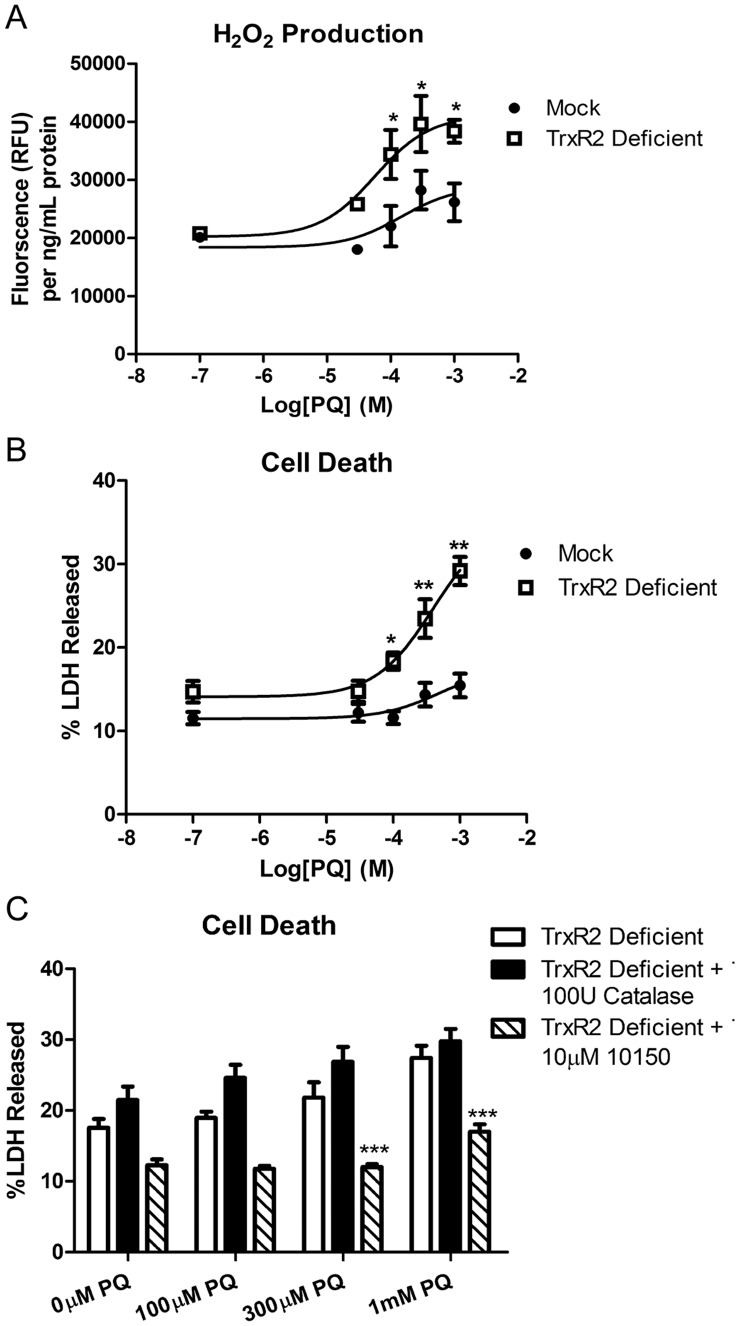
Increased susceptibility to H_2_O_2_ production and cell death in TrxR2 deficient cells. (a) TrxR2 deficient and mock control cells were exposed to varying concentration of PQ for 12 hr and H_2_O_2_ production was measured via Amplex Red. At 100 µM, 300 µM and 1 mM PQ concentrations there was a significant increase in H_2_O_2_ released in the deficient cells compared to mock controls (n = 6) * = p<0.05 as determined 2-way ANOVA. (b) Cell death was determined in mock and TrxR2 deficient cells after 24 hrs exposure to varying concentrations of PQ (n = 12−16). * = p<0.01, ** =  p<0.001 as determined by 2-way ANOVA. (c) TrxR2 deficient cells were exposed to varying concentrations of PQ alone and in combination with the 100 U catalase and 10 µM AEOL10150 for 24 hrs. Catalase was unable to rescue the PQ induced cell death but co-incubation with AEOL10150 was able to significantly decrease %LDH released in TrxR2 deficient cells * = p<0.05, *** = p<0.001 as determined by 2-way ANOVA compared to TrxR2 deficient cells with PQ alone. Data points represent mean ± SEM (n = 6−30).

To determine the role of the mitochondrial Trx/Prx system in controlling steady-state H_2_O_2_ levels and cell viability, TrxR2 deficient and mock control cells were incubated with varying concentrations of PQ and H_2_O_2_ and %LDH release were measured. As indicated in figure 4, a concentration-dependent increase in H_2_O_2_ production occurred in TrxR2 deficient cells compared to mock controls at higher concentrations of PQ (concentrations of 30 µM, 100 µM, 300 µM and 1 mM PQ resulted in 43±5%, 56±19%, 40±17% and 47±7.5% increase in H_2_O_2_ release, respectively). Additionally, 24 hrs of incubation with varying concentrations of PQ resulted in a higher magnitude of LDH release in the TrxR2 deficient cells compared to mock controls. Figure 4b shows a 58±9%, 63.5±16% and 89±11% increase in %LDH released at 100 µM, 300 µM and 1 mM PQ, respectively.

Next, to confirm an intracellular oxidative stress-mediated mechanism of cell death, we asked if the endogenous cell impermeant antioxidant, catalase or a broad spectrum cell-permeable catalytic antioxidant, AEOL10150 [13], [21], [22], inhibited cell death in the TrxR2 deficient cells. As shown in figure 4c, AEOL10150 but not catalase inhibited cell death in TrxR2 deficient cells co-treated with PQ. This data confirmed oxidative stress in the mechanism of cell death and suggested the role of intracellular ROS in the process. With the demonstration that TrxR2 was deficient specifically in the mitochondrial, but not cytosolic compartment, this data suggests a role of excessive mitochondrial H_2_O_2_ in the death of PQ-treated TrxR2 deficient cells. A second lentiviral particle with the sequence, ACAGTTCACGGTGTCGACA was also tested. This resulted in a knocked down of TrxR2 mRNA levels of approximately 30%. With ∼30% knockdown of TrxR2, there was a significant increase in H_2_O_2_ release and PQ toxicity in construct #2 TrxR2 deficient cells at 100, 300 and 1,000 µM PQ with 34, 125 and 127% increase in LDH released respectively (Fig. S2).

### Synergistic Decrease of Mitochondrial Bioenergetics by Combined Treatment of N27 Cells with Aur and PQ

**Figure 5:**
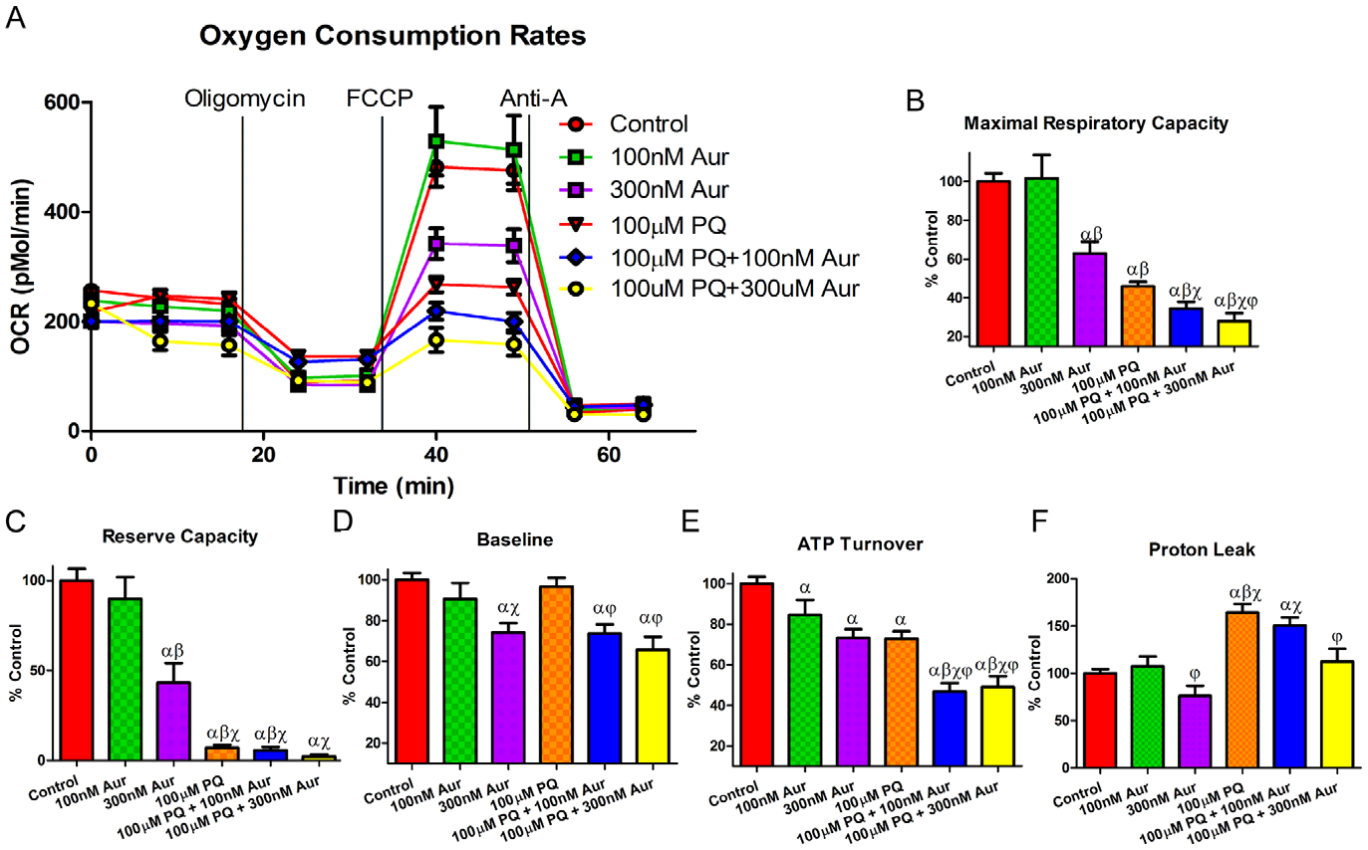
Oxygen Consumption Rates (OCR) and respiration parameters in Aur treated N27 cells. N27 cells were treated with 100 nM or 300 nM Aur alone or in combination with 100 µM PQ for 18 hrs. (a) Oxygen Consumption Rate (OCR) trace was determined using a Seahorse XF24 Analyzer. (b) Maximum Respiratory Capacity (c) Reserve Respiratory Capacity (d) Baseline Respiratory Capacity and (e) ATP Turnover where all decreased in cells treated with Aur and PQ and further decreased with the combined treatments. (e) Proton Leak was increased in cells treated with PQ alone or combined with Aur. α = p<0.05 compared to control, β = p<0.05 compared to 100 nM Aur, χ = p<0.05 compared to 300 nM Aur, φ = p<0.05 compared to 100 µM PQ (n = 5−15) as determined by 1-way ANOVA. Bars represent mean ± SEM.

To determine if the synergistic increase of H_2_O_2_ production and resulting increased cell death in N27 cells occurred as a result of mitochondrial dysfunction, we assessed oxygen consumption rates (OCR) using extracellular flux analysis in cells treated with the PQ and Aur concentrations that generated synergistic increases in figure 3 for 18 hrs. 18 hrs was chosen due to the observation that this time-point corresponded with minimal cell death (LDH release) but increased H_2_O_2_ release (Fig. S3c and d). A bioenergetic (BE) profile of N27 cells treated with Aur in the presence or absence of PQ was generated by injecting electron transport chain (ETC) inhibitors (oligomycin, an ATP synthase inhibitor, FCCP, a mitochondrial uncoupler, and antimycin A (Anti-A), a complex III inhibitor) to probe mitochondria in different states of respiration (Fig. 5a). Pilot experiments showed no change between BE profiles in N27 cells treated with brief exposure to PQ when normalized to protein levels and therefore data is represented as % control of OCR (pmol/min). As indicated in figure 5, combined treatment of PQ and Aur resulted in a decrease maximal respiratory capacity (Fig. 5b), reserve capacity (Fig. 5c) and ATP turnover (Fig. 5d) compared to control and Aur or PQ alone. Additionally, treatment with 300 nM Aur alone or combined treatment of 100 µM PQ with 100 or 300 nM Aur resulted in a decreased baseline OCR in N27 cells (Fig. 5e). Finally, treatment with PQ alone and PQ plus Aur resulted in an increase in mitochondrial proton leak (Fig. 5f). Additionally there was a decrease in cellular ATP levels in 300 nM Aur and 100 µM PQ +300 nM Aur compared to control and no change in a subunit of mitochondrial complex IV (Cox IV) levels (Fig. S4a and S5b). This further supports the role of mitochondrial dysfunction in N27 cells. Consistent with the H_2_O_2_ release and cell death, these data suggest that the combination of TrxR inhibition and oxidative stress and not either treatment alone is sufficient to decreased maximal and spare mitochondrial OCR prior to loss of cell viability.

### TrxR2 Deficiency Decreases Mitochondrial Respiration in N27 Cells

**Figure 6:**
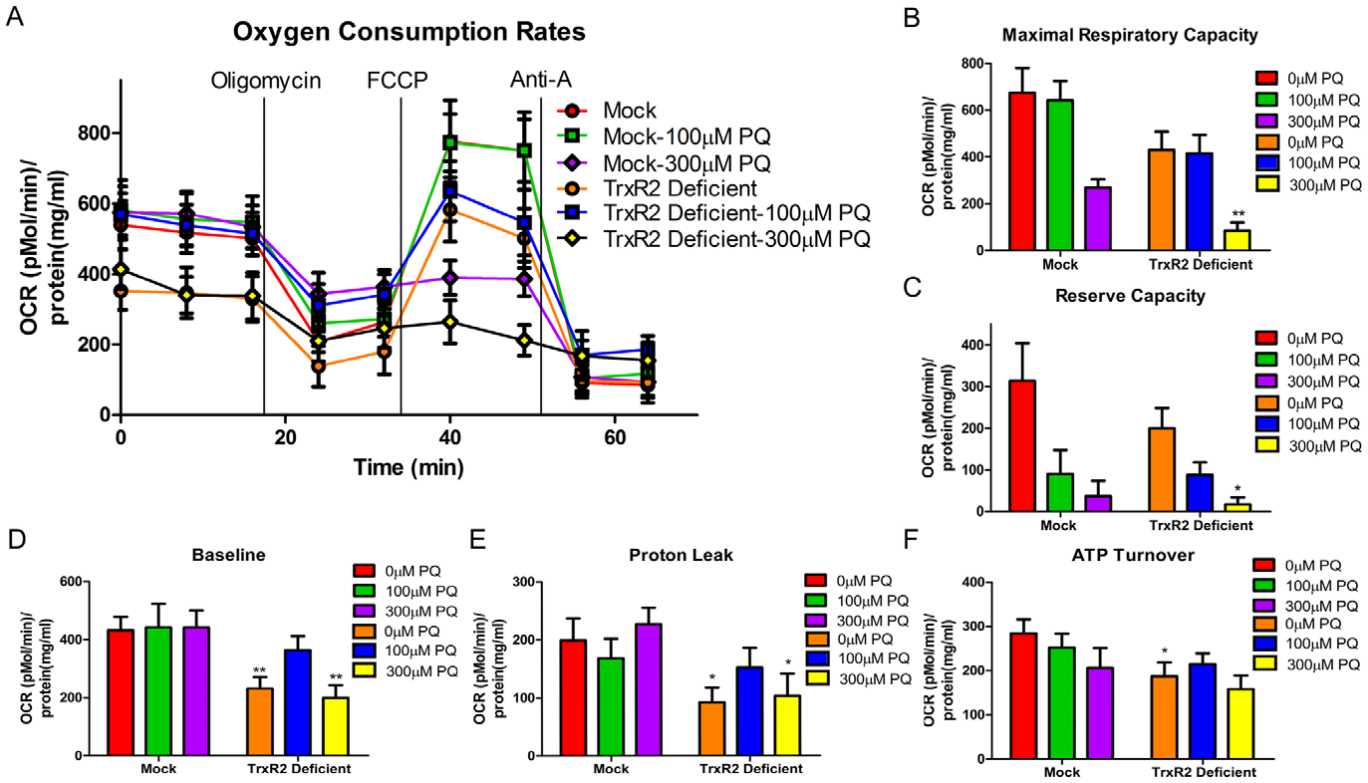
Oxygen Consumption Rates (OCR) and respiration parameters in mock control and TrxR2 deficient cells. Stably transfected cells were treated with 100 µM or 300 µM PQ for 6 hrs. (a) Oxygen Consumption Rate (OCR) trace was determined using a Seahorse XF24 Analyzer. (b) Maximal Respiratory Capacity (c) Reserve Respiratory Capacity (d) Baseline Respiratory Capacity and (f) Proton leak where all decreased in cells treated with 300 µM PQ. (e) ATP turnover was decreased in TrxR2 deficient cells with no PQ treatment compared to mock control. * = p<0.05 compared to mock control with same PQ concentration treatment. ** = p<0.01 mock control with same PQ concentration treatment (n = 7−9) as determined by two-tailed students t-test. Bars represent mean ± SEM.

We further asked whether mitochondrial dysfunction occurred in TrxR2 deficient cells in the presence or absence of 100 or 300 µM PQ for 6 hrs. The 6 hr time-point was chosen due to the observation that the knockout cells have increased H_2_O_2_ release and but no cell death (Fig. S3a and b). Thus to determine OCR rates prior to a loss of cell viability, the 6 hr time point was chosen. Additionally, N27 cells treated with Aur and PQ for 6 hrs had a decrease in maximal, reserve and baseline respiration compared to control (Fig. S6). To rule out any effects generated due to different growth rates or protein levels between 2 different cell types, OCR levels were normalized to protein levels (pmol/min/mg protein). As shown in figure 6a, different mitochondrial respiration parameters were determined based on the OCR after being subjected to different ETC inhibitors. There was a decrease in baseline (Fig. 6d), ATP turnover (Fig. 6e) and proton leak (Fig. 6f) between the mock and TrxR2 deficient cells with no PQ treatment. Additionally after 6 hrs of treatment with 300 µM PQ there was a decrease in maximal respiratory capacity (Fig. 6b), reserve capacity (Fig. 6c), baseline respiration (Fig. 6d) and proton leak (Fig. 6f). We also observed decrease in overall maximal respiratory capacity and spare respiratory capacity by 300 µM PQ compared to the mock or deficient controls which was exacerbated in TrxR2 deficient cells. Interestingly, mock transfected cells had a decrease in maximal and spare respiratory capacity at 100 µM PQ similar to TrxR2 deficient cells indicating PQ has a general effect on mitochondrial respiration (Fig. 6B and C). However at a slightly higher concentration (300 µM) the respiratory defects in the mock cells did not change, but there was a statistically significant decrease in the TrxR2 deficient cells. This suggests that PQ treatment will cause a general decrease, but with the loss of TrxR2 a slightly higher concentration, which is still non-toxic in control cells, will exacerbate cell death and maximal and spare respiratory capacity. No changes were observed in glycolytic rates (data not shown). Together this data suggests that TrxR2 deficient cells display lower basal OCR rates, ATP turnover and proton leak but no difference in maximal OCR. However, exposure of TrxR2 deficient cells to PQ exacerbated the decrease observed in maximal OCR but not other parameters.

### 6OHDA Replicates the Effects of PQ

**Figure 7:**
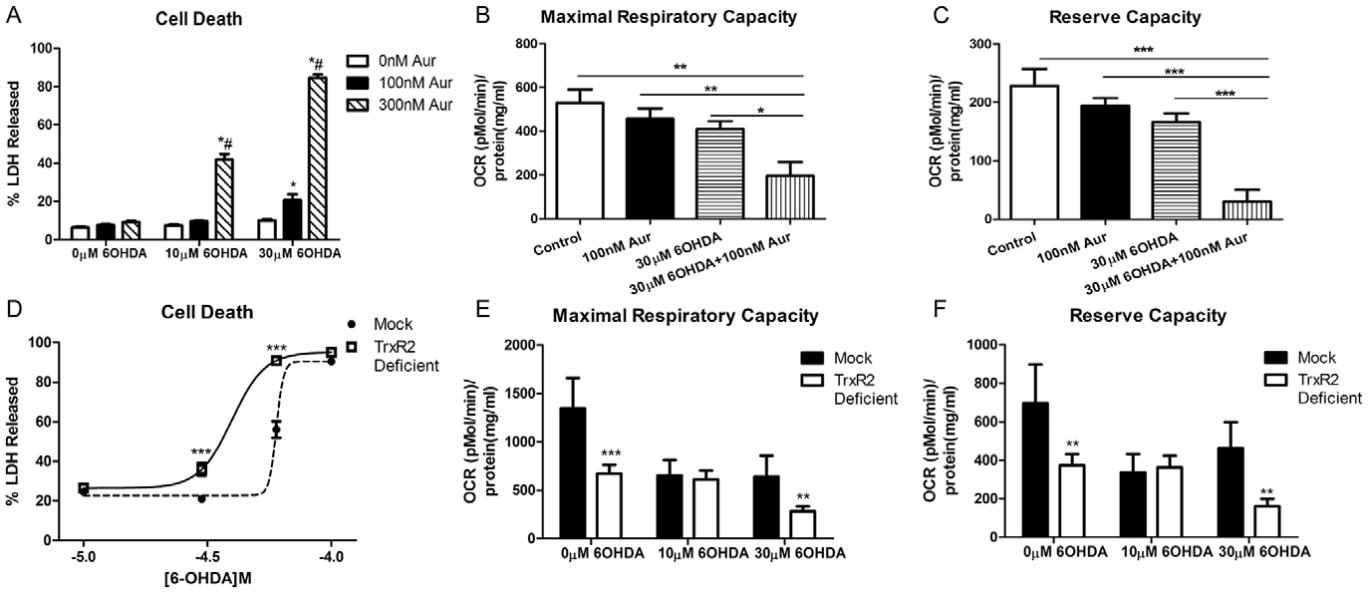
Effects of Aur treated or TrxR2 deficient N27 cells with 6OHDA. (a) N27 cells were exposed to varying concentration of 6OHDA and Aur for 24 hr and cell death was measured via percent LDH released (n = 8−16). * = p<0.001 compared to 0 nM Aur with same 6OHDA concentration; # = p<0.001 compared to 100 nM Aur with same 6OHDA concentration determined by 2-way ANOVA. (b) Maximal respiratory capacity and (c) Reserve respiratory capacity were measured with an XF analyzer after 18 hr incubation (n = 4−5) * = p<0.05, ** = p<0.01 as determined by 1-way ANOVA. (d) TrxR2 mock and deficient cells were treated with varying concentrations of 6OHDA and cell death was measured (n = 6−12). *** = p<0.001 as determined by 1-way ANOVA. (e) After 6 hr incubation with 6OHDA there was a significant decrease in both maximal respiratory capacity (e) and reserve capacity (f) in TrxR2 deficient cells alone and treated with 30 µM 6OHDA (n = 7−11) determined by two-tailed students t-test ** = p<0.005, *** = p<0.001. Bars represent mean ± SEM.

To determine if the observations above were limited to PQ, N27 cells treated with Aur and TrxR2 deficient cells were exposed to varying concentration of the known parkinsonism toxin 6-hydroxydopamine (6OHDA). 6OHDA is a redox cycling catecholamine analogue that accumulates in the terminals of monoaminergic neurons and induces neuronal degeneration [11], [12]. After 18 and 24 hrs of exposure, OCR and cell death was measured in both cells types, respectively. As shown in 7b and 7c incubation with both Aur and 6OHDA results in a synergistic decrease in maximal and reserve respiratory capacities in N27 cells compared to either compound alone. Additionally after 24 hrs of exposure there is a significant increase in cell death in N27 cells with combined treatment compared to either compound alone (Fig. 7a). Similarly, compared to mock controls, TrxR2 deficient cells showed synergistic decreases in maximal respiratory capacity and reserve respiratory capacity (Fig. 7e and f) and there was a significant shift in cell death following 6OHDA exposure (Fig. 7d). Consistent with PQ, mock transfected cells had a decrease in maximal and spare respiratory capacities at 10 µM 6OHDA with no further decrease at 30 µM. There was a statistically significant decrease in the TrxR2 deficient cells at 30 µM compared to mock controls. This suggests that loss of TrxR2 exacerbates 6OHDA-induced cell death and maximal as well as spare respiratory capacity. In sum, 6OHDA treatment closely mimicked the effects of PQ suggesting the importance of the mitochondrial Trx/Prx system in controlling oxidative stress in response to multiple parkinsonian toxicants.

## Discussion

Here we demonstrate a critical role of the mitochondrial Trx/Prx system in oxidative stress-mediated neuronal death by parkinsonian toxicants. Three principal findings emerge from this work. First, sub-toxic oxidative stress concomitant with pharmacological inhibition of TrxR in primary mesencephalic cultures and N27 dopaminergic cells results in a synergistic increase in H_2_O_2_ release and potentiation of cell death indicating the importance of the Trx/Prx pathway in H_2_O_2_ detoxification. Secondly, TrxR2 deficiency renders N27 cells more susceptible to H_2_O_2_ release and subsequent cell death following subtoxic oxidative stress confirming that mitochondrial specific Trx/Prx pathway plays a critical role in H_2_O_2_ detoxification and oxidative stress-mediated cell death. Finally, constitutive TrxR2 deficiency results in deficits in mitochondrial respiratory parameters. TrxR2 deficiency exacerbates the decrease in maximal OCR and spare respiratory capacity in the presence of sub-toxic oxidative stress. Together, these results highlight the importance of the mitochondrial Trx/Prx pathway in H_2_O_2_ detoxification and consequent cell death in response to parkinsonian toxicants.

Current PD research has focused on the formation of ROS leading to oxidative damage and ultimately DA loss within the substantia nigra pars compacta (SNpc) [2], [3], [23]. There are 2 major known antioxidant systems in brain mitochondria for detoxifying ROS, the glutathione system and the Trx/Prx system. We recently showed that isolated brain mitochondria are not only major producers of H_2_O_2_ [9] but they are also capable of consuming H_2_O_2_ in a substrate- and respiration-dependent manner predominantly via the Trx/Prx system [8]. Since H_2_O_2_ at low levels plays an important role in cell signaling, merely compromising all H_2_O_2_ detoxification may not serve a beneficial role [1], [24], [25]. The precedence for age-related decline of antioxidant systems in neurodegeneration comes from findings demonstrating decreased antioxidant levels and altered ability to up regulate antioxidant levels in older animals and humans [26], [27]. An age-dependent decrease in hippocampal TrxR activity in humans with Alzheimer’s disease (AD) [28] suggests an association with age-related antioxidant function leading to oxidative stress. With respect to PD, it was revealed that with increasing age there is a decrease in superoxide dismutase (SOD), glutathione peroxidase (GPx) and glutathione reductase (GR) in the substantia nigra (SN) but there was no change in GPx and GR in the caudate nucleus with increasing age [18] [29]. Interestingly, there was an increase in TrxR activity in the same areas independent of aging [29]. Additionally, proteomic analysis of human SN in PD tissue showed an increase in Prx2, complex III and ATP synthase in PD patients compared to control [30]. This data is suggestive of alterations in mitochondrial ROS scavenging proteins in aging and neurodegenerative diseases. However, a clear resolution regarding the importance of mitochondrial H_2_O_2_ scavenging systems in mediating neurodegeneration is needed.

The significance of the mitochondrial H_2_O_2_ removal by the Trx/Prx system in rendering neuronal cells vulnerable to mild oxidative stress mediated by parkinsonian toxicants demonstrated in this study is supported by two lines of evidence. First, pharmacological inhibition by Aur at subtoxic concentrations potentiated H_2_O_2_ release and cell death resulting from subtoxic concentrations of PQ, in two cell types, the mesencephalic primary cultures and the N27 dopaminergic cells. This result is consistent with our previous work in isolated mitochondria indicating the role of the Trx/Prx system in H_2_O_2_ consumption. Furthermore, this suggests that loss of cell viability occurs only when both oxidative stress occurs and a major antioxidant defense i.e. the Trx/Prx system is compromised.

A second line of evidence (shRNA mediated inhibition of the TrxR2) confirmed the effects observed with pharmacological inhibition using Aur. Trx and TrxR are generated in 3 isoforms, Trx1/TrxR1 which is located in the cytosol, Trx2/TrxR2 located in the mitochondria and Trx3/TrxR3 located in the testis [20]. It has been shown that reduced Trx1 inhibits the activity of apoptosis signal-regulated kinase-1 (ASK1) and inhibition of TrxR1 can lead to the disassociation of Trx1 with ASK1 and activation of the apoptotic pathway [31]. The Trx2/TrxR2 system uses a similar pathway for H_2_O_2_ detoxification as Trx1/TrxR1, however, Trx1 has 3 more cysteine residues than Trx2 leading the mitochondrial form to have a higher resistance to oxidation due to dimer formation of Trx1 when the additional cysteine residues are oxidized leading to a loss of catalytic activity [20], [32]. Previous literature has demonstrated that over-expression of Trx2 renders cells more resistant to cell death via oxidative stress and can increase the mitochondrial membrane potential [32], [33]. Additionally, deletion of Trx2 and TrxR2 via siRNA renders cells more susceptible to apoptotic stimuli in endothelial cells, myoblasts and cardiomyocytes [7], [34], [35]. Knockout of Trx2 and TrxR2 in a mouse model is embryonically lethal at day 10.5 and 13 respectively and the timing of this lethality coincides with the maturation of mitochondria [6], [36]. Interestingly, heterozygous Trx2 mice appear normal, however they have increased levels of ROS production, increased oxidative damage, and an increase in sensitivity to diquat exposure compared to homozygous Trx2 mice [37]. The specific knockdown of TrxR2 did not alter TrxR1 expression, however, we did not specifically determine whether expression levels of other cellular antioxidant enzymes were altered to partially compensate for TrxR2 deficiency.

Our ability to demonstrate increased susceptibility to oxidative stress in TrxR2 deficient N27 cells provides the first evidence of the importance of the mitochondrial thioredoxin pathway in neuronal cells. Moreover, inhibition of PQ-induced cell death in TrxR2 deficient cells by a cell permeant catalytic antioxidant, AEOL10150, but not the cell impermeant antioxidant catalase confirms oxidative stress in the mechanism of cell death via intracellular ROS production. AEOL10150 is a catalytic antioxidant with a potent and wide spectrum of activity against superoxide, H_2_O_2_, peroxynitrite, and lipid peroxyl radicals [13]. Protection of vulnerable targets such as mitochondrial aconitase suggest that metalloporphyrins such as AEOL10150 and closely related compound, AEOL10113, can target mitochondrial superoxide [38], [39]. In fact, over expression of manganese superoxide dismutase and metalloporphyrins such as MnTBAP and AEOL10113 have previously been shown to protect against PQ or 6OHDA toxicity [18], [40]–[42].

Assessment of mitochondrial bioenergetics following pharmacological inhibition with Aur or TrxR2 deficient cells revealed both a predictable and a surprising finding. First, based on the above studies we found that bioenergetic parameters were also potentiated following Aur and subtoxic PQ treatment. Our data showed that combined treatment with Aur and PQ rendered the reserve capacity close to zero indicating the cells were operating at their bioenergetic limit and that there was limited spare respiratory capacity as a result. These results explain the increased H_2_O_2_ production and cell death observed in figure 2b and c. PQ alone decreased the BE profile but cells were able to compensate for the increased H_2_O_2_ production via the Trx/Prx pathway resulting in a lack of overt cell death. However with combined incubation, cells lose a major cellular defense against H_2_O_2_ resulting in an inability of mitochondria to cope with the onslaught of oxidative stress. As a result, a synergistic H_2_O_2_ release and cell death ensued.

Surprisingly, constitutive deficiency of the mitochondrial specific TrxR2 decreased all BE parameters, including basal respiration which was not observed in the pharmacological inhibition. This may be due to the prolonged constitutive nature of TrxR2 deficiency resulting in an inability to handle even constitutive levels of H_2_O_2_. This is supported by our previous work demonstrating the importance of the mitochondrial Trx/Prx in H_2_O_2_ removal [8] and data by Pérez et al looking at Trx2 heterozygous mice (Trx2^+/−^) which had increased macromolecule oxidation under normal conditions [37].

The demonstration that 6OHDA, a well established parkinsonian toxicant replicates the findings observed with PQ suggests that endogenous and exogenous oxidants potentiate cell death under conditions with compromised mitochondrial antioxidant defenses. Multiple other compounds and pesticides have been implicated in development of sporadic PD such as rotenone, 1-methyl-4-phenyl-1,2,3,6-tetrahydropyride (MPTP) and 6-hydroxydopamine (6OHDA) [12], [43]. 6OHDA uses the catecholamine transporter system to enter into DA cells and generates O_2_^._^ and H_2_O_2_ from its autooxidation reaction with oxygen to form toxic quinones that inhibit ETC complexes [43]–[45]. Non-toxic treatment with 6OHDA to Aur treated and TrxR2 deficient N27 cells resulted in increased cell death and decreased maximal and spare respiratory capacity compared to either group alone. This data suggests that the role of the mitochondrial Trx/Prx system to maintain mitochondrial bioenergetics under non-toxic levels of oxidative stress is not specific to the action of PQ, but a general mechanism.

In sum, this study demonstrates a crucial role of mitochondrial TrxR2 in the maintenance of mitochondrial function, steady-state H_2_O_2_ levels and neuronal cell death. To our knowledge this is the first study to unequivocally implicate the mitochondria-specific TrxR2 in neurotoxicity by creation of a stable neuronal cell line with TrxR2 deficiency. The major finding that sub-toxic oxidative stress concomitant with deficiency of TrxR2 results in a potentiation of H_2_O_2_ release and cell death indicates the importance of the Trx/Prx pathway. This supports the “two-hit” model of neurodegeneration in which ongoing oxidative stress associated with aging or genetic predisposition synergizes with environmental factors.

## Supporting Information

Figure S1 **Pharmacological inhibition of TrxR for 24 hr in primary mesencephalic cultures and N27 cells results in increased cell death.** (a) subtoxic concentration of Aur or PQ caused a minimal increase in cell death after 24 hrs however combined treatment resulted in a significant increase in %LDH released in primary mesencephalic cultures (n  = 12−16). (b) In N27 cell, combined incubation of sub-toxic concentrations of PQ and Aur for 24 hrs resulted in no increase in %LDH released with individual treatment but a significant increase with 300 nM Aur combined with both concentrations of PQ (n = 10−15). Bars represent mean ± SEM. α =  p<0.05 compared to 0 nM Aur in same PQ treatment, β = p<0.05 compared to 100 nM Aur in same PQ treatment, χ = p<0.05 compared to 0 µM PQ in same Aur treatment, φ = p<0.01 compared to 100 µM PQ in same Aur treatment as determined by 2-way ANOVA.
(TIF)

Figure S2 **shRNA construct #2 generated a loss of TrxR2 mRNA and an increase in H2O2 production and subsequent cell death.** shRNA construct #2 was transfected in N27 cells as outlined in the materials and methods and resulted in a 30% decrease in mRNA levels compared to mock control. * = p<0.05 by student’s t-test. After 12 (b) and 24 (c) of incubation with varying concentrations of PQ there was a significant increase in H_2_O_2_ production and cell death compared to mock controls. * = p<0.05, ** = p<0.001 as determined by 2-way ANOVA.
(TIF)

Figure S3 **Cell death and H_2_O_2_ production at BE profile time points for N27, Mock and TrxR2 deficient cells.** (a) After 6 hr of incubation with varying concentrations of PQ there was no change in cell death in both Mock and TrxR2 deficient cells and a significant increase in H_2_O_2_ production in both cell types (b). ** = p<0.01 and *** = p<0.001 (n = 6). N27 cells incubated with Aur and PQ alone or in combination had a significant increase in cell death only in the 300 nM Aur +300 µM PQ group (c) (*** = p<0.001) while there was significant increase in H_2_O_2_ production in 300 nM Aur and all combined treatments (d). Bars represent mean ± SEM (n-4-12) α =  p<0.05 compared to 0 nM Aur in same PQ treatment, β = p<0.05 compared to 100 nM Aur in same PQ treatment, χ = p<0.05 compared to 0 µM PQ in same Aur treatment, φ = p<0.01 compared to 100 µM PQ in same Aur treatment as determined by 2-way ANOVA.
(TIF)

Figure S4 **ATP Levels in N27, Mock and TrxR2 deficient cells.** (a) N27 cells were treated with 100 or 300 nM Aur, 100 µM PQ or a combination for 18 hr. There was a significant decrease in ATP levels in the 300 and 100 µM PQ +300 nM Aur group, 100 nM Aur and 100 µM PQ compared to control as determined by 1-way ANOVA (** = p<0.001). In mock transfected cells (b) treated with varying concentration of PQ there was no change in ATP levels after 6 hrs of treatment. TrxR2 deficient cells treated for 6 hr (c) had a significant decrease in ATP levels compared to control as determined by student’s T-test (* = p<0.05).
(TIF)

Figure S5 **Complex IV levels in N27, Mock and TrxR2 deficient cells measured via western blot. (**a) Representative blot of actin and complex IV in N27 cells treated for 18 hr with 100 µM PQ or 100 nM Aur. (b) Complex IV levels in N27 cells treated with Aur and PQ alone and in combination (n = 3). (c) Mock and TrxR2 deficient cells were treated with 100 or 300 µM PQ for 6 hrs and Cox IV levels were measured and normalized to actin levels (n = 3). There was no change in Cox IV levels in any treatment group to control levels.
(TIF)

Figure S6 **Oxygen Consumption Rates (OCR) and respiration parameters in Aur treated N27 cells for 6** **hrs.** N27 cells were treated with 100 nM or 300 nM Aur alone or in combination with 100 µM PQ for 6 hrs. (a) Oxygen Consumption Rate (OCR) trace was determined using a Seahorse XF24 Analyzer. (b) Maximum Respiratory Capacity (c) Reserve Respiratory Capacity (d) Baseline Respiratory Capacity and (e) ATP Turnover where all decreased in cells treated with Aur and PQ and further decreased with the combined treatments. (e) Proton Leak was increased in cells treated with PQ alone. The results obtained after 6 hrs parallel the results obtained after 18 hrs (figure 5) however to a lesser extent. α = p<0.05 compared to control, β = p<0.05 compared to 100 nM Aur, χ = p<0.05 compared to 300 nM Aur, φ = p<0.05 compared to 100 µM PQ (n = 5−20) as determined by 1-way ANOVA. Bars represent mean ± SEM. The results obtained after 6 hrs parallel the results obtained after 18 hrs (figure 5) however to a lesser extent.
(TIF)
