# Supplementary figures and images for: Thioredoxin Reductase Deficiency Potentiates Oxidative Stress, Mitochondrial Dysfunction and Cell Death in Dopaminergic Cells
Source: PLoS One. 2012 Nov 30;7(11):e50683. doi: 10.1371/journal.pone.0050683 (PMC3511321; doi:10.1371/journal.pone.0050683)

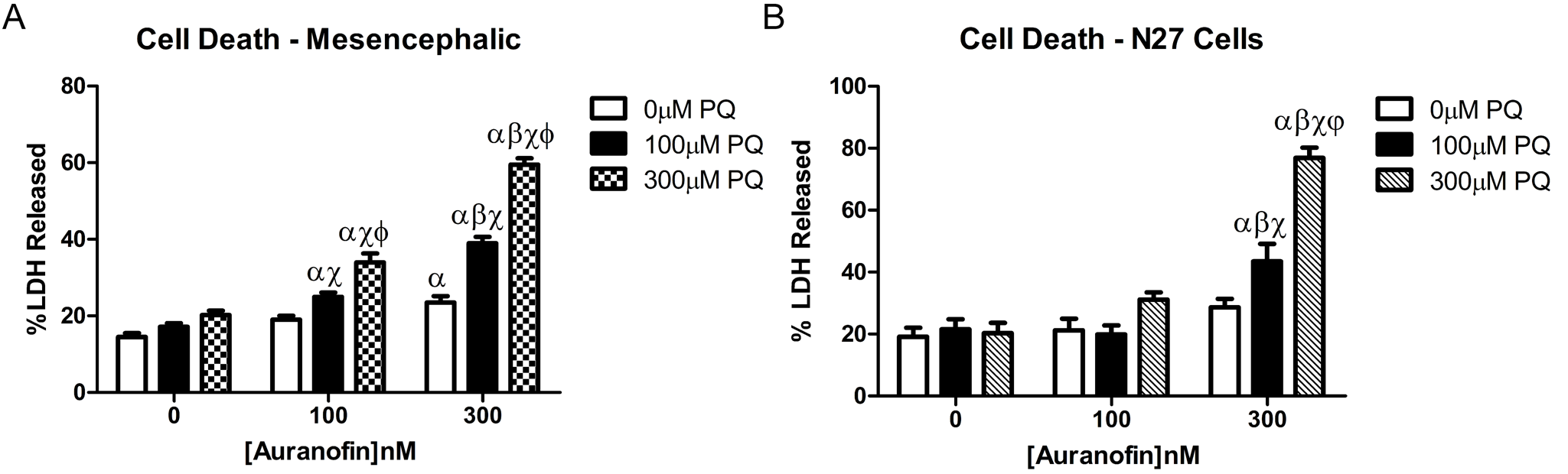

Supplement: Figure S1 — Pharmacological inhibition of TrxR for 24 hr in primary mesencephalic cultures and N27 cells results in increased cell death. (a) subtoxic concentration of Aur or PQ caused a minimal increase in cell death after 24 hrs however combined treatment resulted in a significant increase in %LDH released in primary mesencephalic cultures (n = 12−16). (b) In N27 cell, combined incubation of sub-toxic concentrations of PQ and Aur for 24 hrs resulted in no increase in %LDH released with individual treatment but a significant increase with 300 nM Aur combined with both concentrations of PQ (n = 10−15). Bars represent mean ± SEM. α = p<0.05 compared to 0 nM Aur in same PQ treatment, β = p<0.05 compared to 100 nM Aur in same PQ treatment, χ = p<0.05 compared to 0 µM PQ in same Aur treatment, φ = p<0.01 compared to 100 µM PQ in same Aur treatment as determined by 2-way ANOVA. (TIF) [file pone.0050683.s001.tif]

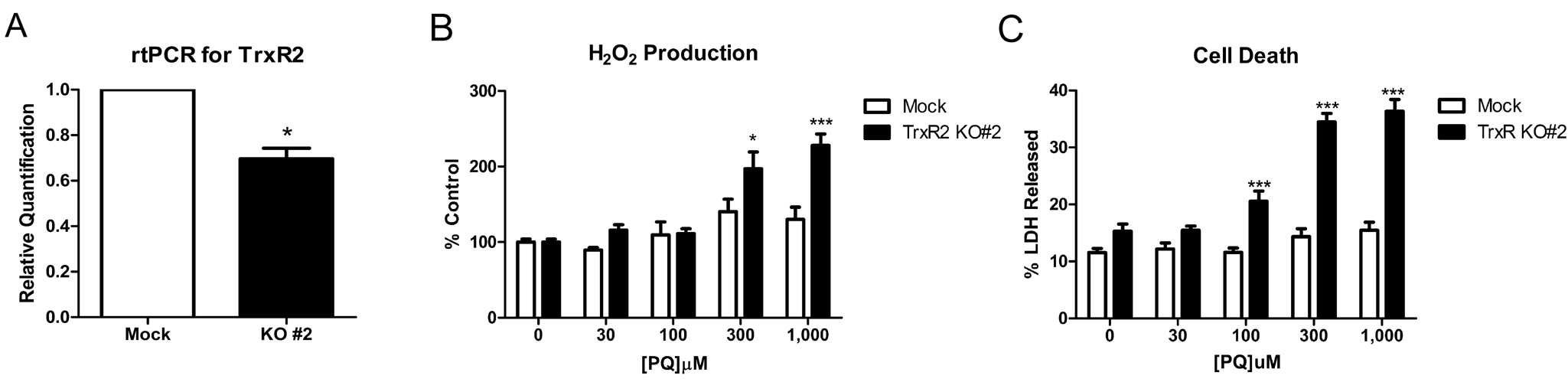

Supplement: Figure S2 — shRNA construct #2 generated a loss of TrxR2 mRNA and an increase in H2O2 production and subsequent cell death. shRNA construct #2 was transfected in N27 cells as outlined in the materials and methods and resulted in a 30% decrease in mRNA levels compared to mock control. * = p<0.05 by student’s t-test. After 12 (b) and 24 (c) of incubation with varying concentrations of PQ there was a significant increase in H2O2 production and cell death compared to mock controls. * = p<0.05, ** = p<0.001 as determined by 2-way ANOVA. (TIF) [file pone.0050683.s002.tif]

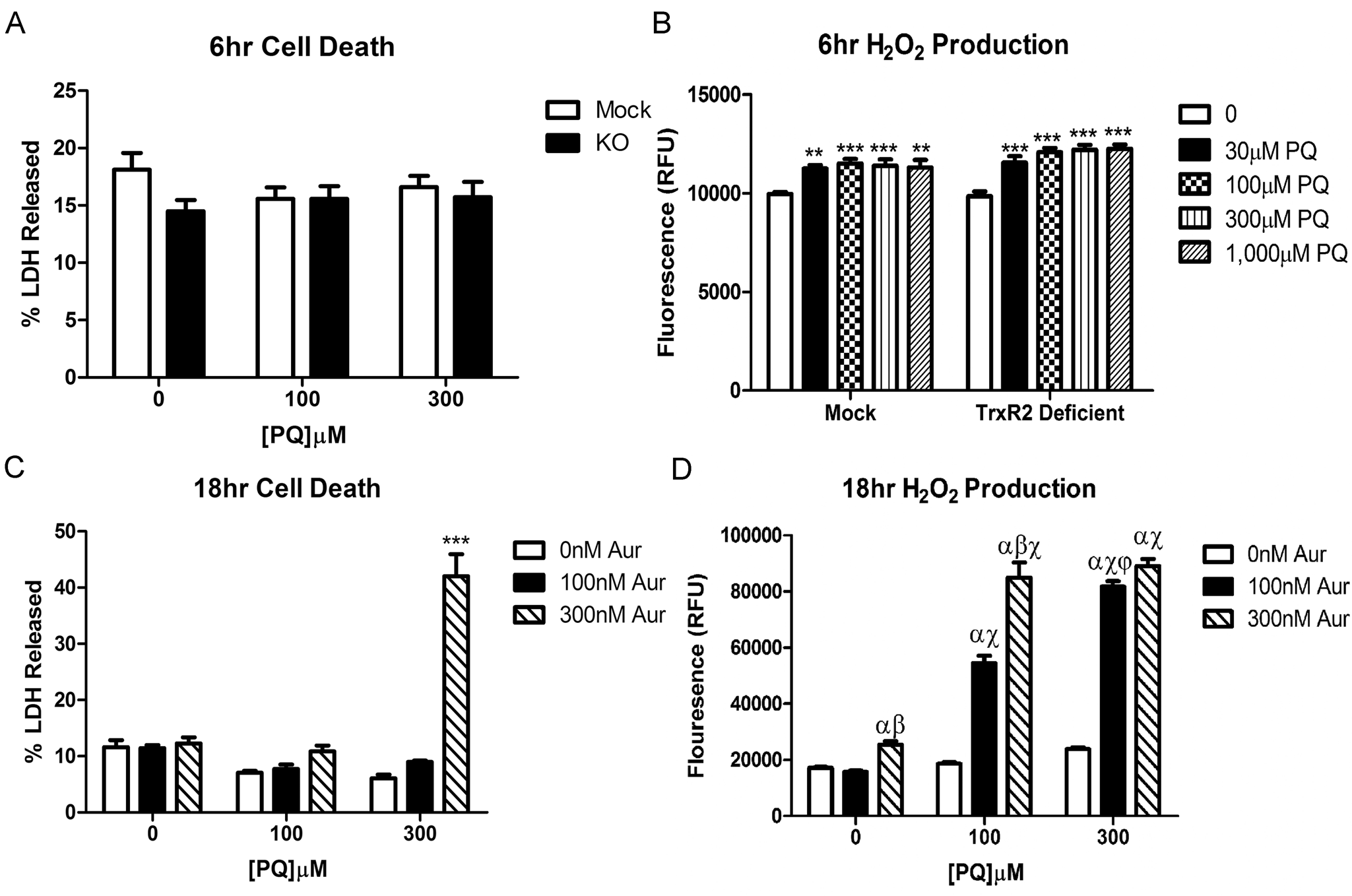

Supplement: Figure S3 — Cell death and H2O2 production at BE profile time points for N27, Mock and TrxR2 deficient cells. (a) After 6 hr of incubation with varying concentrations of PQ there was no change in cell death in both Mock and TrxR2 deficient cells and a significant increase in H2O2 production in both cell types (b). ** = p<0.01 and *** = p<0.001 (n = 6). N27 cells incubated with Aur and PQ alone or in combination had a significant increase in cell death only in the 300 nM Aur +300 µM PQ group (c) (*** = p<0.001) while there was significant increase in H2O2 production in 300 nM Aur and all combined treatments (d). Bars represent mean ± SEM (n-4-12) α = p<0.05 compared to 0 nM Aur in same PQ treatment, β = p<0.05 compared to 100 nM Aur in same PQ treatment, χ = p<0.05 compared to 0 µM PQ in same Aur treatment, φ = p<0.01 compared to 100 µM PQ in same Aur treatment as determined by 2-way ANOVA. (TIF) [file pone.0050683.s003.tif]

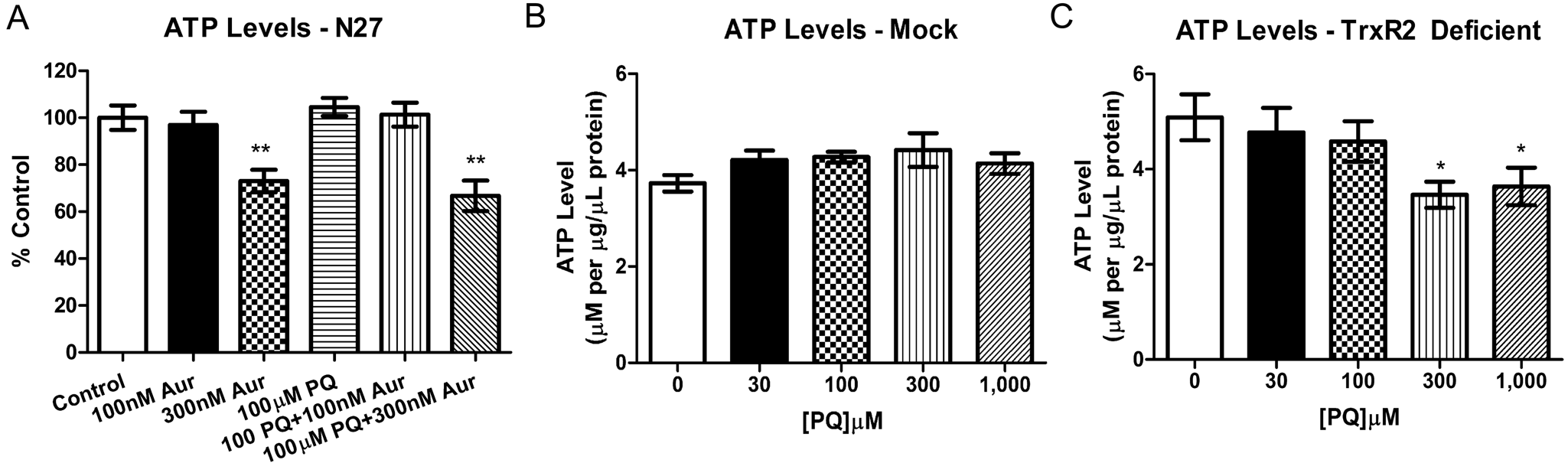

Supplement: Figure S4 — ATP Levels in N27, Mock and TrxR2 deficient cells. (a) N27 cells were treated with 100 or 300 nM Aur, 100 µM PQ or a combination for 18 hr. There was a significant decrease in ATP levels in the 300 and 100 µM PQ +300 nM Aur group, 100 nM Aur and 100 µM PQ compared to control as determined by 1-way ANOVA (** = p<0.001). In mock transfected cells (b) treated with varying concentration of PQ there was no change in ATP levels after 6 hrs of treatment. TrxR2 deficient cells treated for 6 hr (c) had a significant decrease in ATP levels compared to control as determined by student’s T-test (* = p<0.05). (TIF) [file pone.0050683.s004.tif]

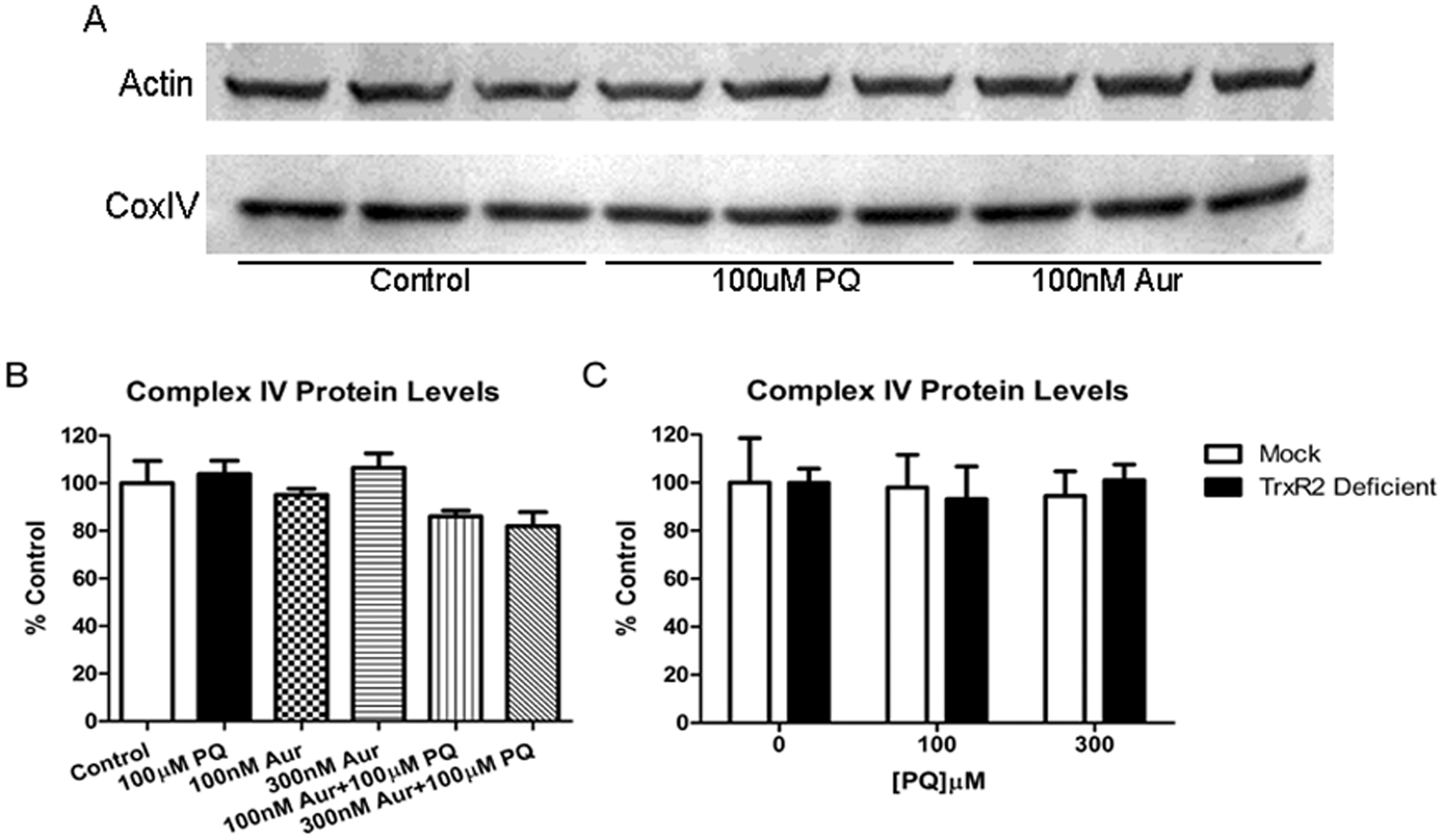

Supplement: Figure S5 — Complex IV levels in N27, Mock and TrxR2 deficient cells measured via western blot. (a) Representative blot of actin and complex IV in N27 cells treated for 18 hr with 100 µM PQ or 100 nM Aur. (b) Complex IV levels in N27 cells treated with Aur and PQ alone and in combination (n = 3). (c) Mock and TrxR2 deficient cells were treated with 100 or 300 µM PQ for 6 hrs and Cox IV levels were measured and normalized to actin levels (n = 3). There was no change in Cox IV levels in any treatment group to control levels. (TIF) [file pone.0050683.s005.tif]

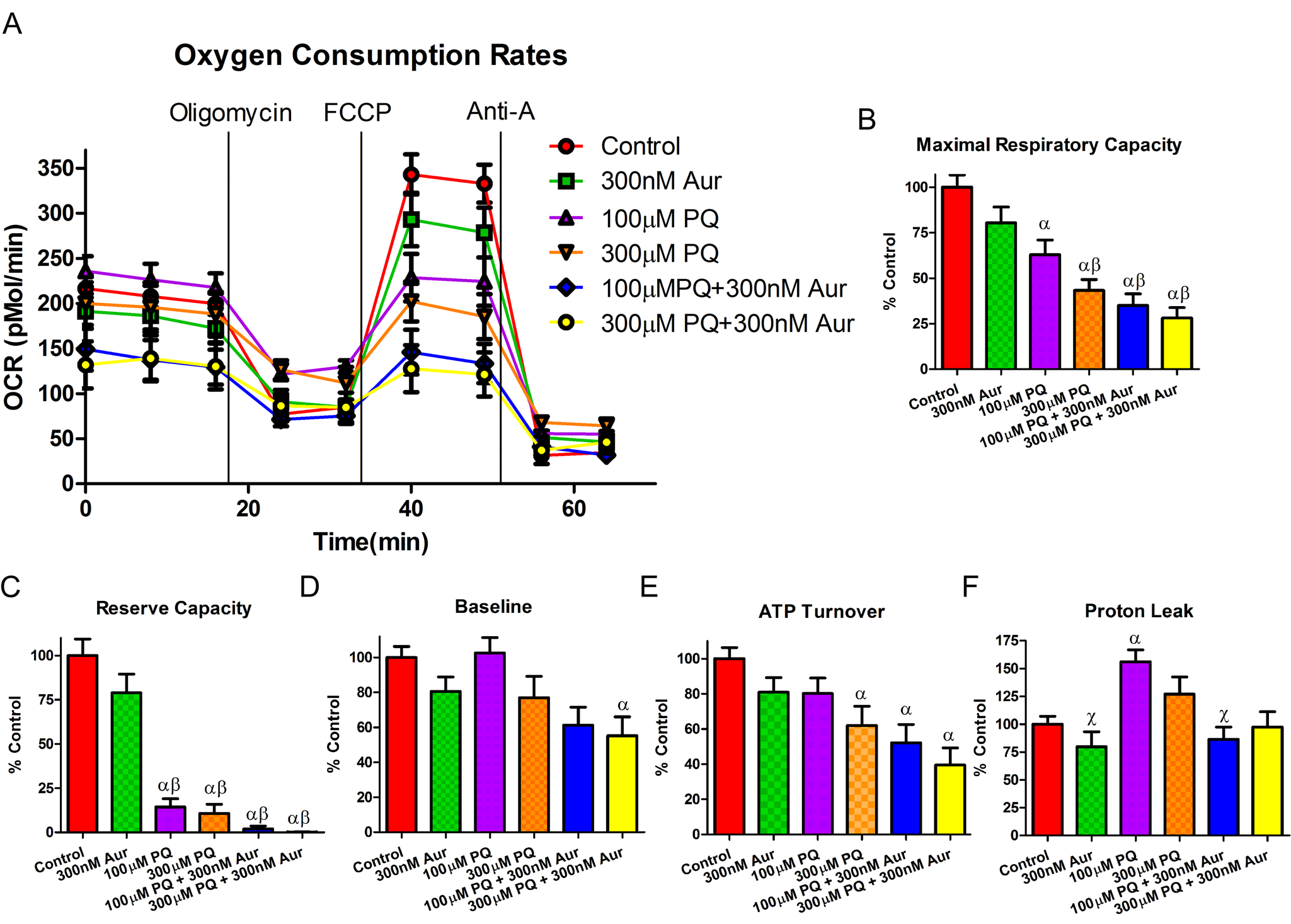

Supplement: Figure S6 — Oxygen Consumption Rates (OCR) and respiration parameters in Aur treated N27 cells for 6 hrs. N27 cells were treated with 100 nM or 300 nM Aur alone or in combination with 100 µM PQ for 6 hrs. (a) Oxygen Consumption Rate (OCR) trace was determined using a Seahorse XF24 Analyzer. (b) Maximum Respiratory Capacity (c) Reserve Respiratory Capacity (d) Baseline Respiratory Capacity and (e) ATP Turnover where all decreased in cells treated with Aur and PQ and further decreased with the combined treatments. (e) Proton Leak was increased in cells treated with PQ alone. The results obtained after 6 hrs parallel the results obtained after 18 hrs (figure 5) however to a lesser extent. α = p<0.05 compared to control, β = p<0.05 compared to 100 nM Aur, χ = p<0.05 compared to 300 nM Aur, φ = p<0.05 compared to 100 µM PQ (n = 5−20) as determined by 1-way ANOVA. Bars represent mean ± SEM. The results obtained after 6 hrs parallel the results obtained after 18 hrs (figure 5) however to a lesser extent. (TIF) [file pone.0050683.s006.tif]
